# Evidence for ecological speciation via a host shift in the holly leaf miner, *Phytomyza glabricola* (Diptera: Agromyzidae)

**DOI:** 10.1002/ece3.2358

**Published:** 2016-08-23

**Authors:** Julie B. Hébert, Sonja J. Scheffer, David J. Hawthorne

**Affiliations:** ^1^ BEES ProgramUniversity of Maryland4112 Plant Science BuildingCollege ParkMaryland20742; ^2^ Systematic Entomology LaboratoryUSDA‐ARS10300 Baltimore Av.BeltsvilleMaryland20705; ^3^ Department of EntomologyUniversity of Maryland4112 Plant Science BuildingCollege ParkMaryland20742

**Keywords:** Amplified fragment length polymorphism, host forms, host races, *Ilex*, sympatric speciation

## Abstract

Evolutionary radiations have been well documented in plants and insects, and natural selection may often underly these radiations. If radiations are adaptive, the diversity of species could be due to ecological speciation in these lineages. Agromyzid flies exhibit patterns of repeated host‐associated radiations. We investigated whether host‐associated population divergence and evidence of divergent selection exist in the leaf miner *Phytomyza glabricola* on its sympatric host plants, the holly species, *Ilex coriacea* and *I. glabra*. Using AFLPs and nuclear sequence data, we found substantial genetic divergence between host‐associated populations of these flies throughout their geographic range. Genome scans using the AFLP data identified 13 loci under divergent selection, consistent with processes of ecological speciation. EF‐1*α* data suggest that *I. glabra* is the original host of *P. glabricola* and that *I. coriacea* is the novel host, but the AFLP data are ambiguous with regard to directionality of the host shift.

## Introduction

Phytophagous insects are extremely diverse, making up over 25% of the total terrestrial biodiversity (Strong et al. 1984; Price 2008). In many groups of these insects, much of the diversity appears to have been generated by an evolutionary escape–and–radiate scenario where a shift to a phylogenetically distant plant species is followed by rapid speciation onto relatively more closely related plant species (Ehrlich and Raven 1964; Mitter et al. 1988; Farrell 1998; Winkler and Mitter 2008). The evolution of key innovations, for example, the ability to digest novel plant defensive chemicals, may provide the primary ecological opportunities needed for the first host shift, yet it is the subsequent host‐associated radiations onto many closely related hosts that give rise to the high levels of biodiversity (Simpson 1949, 1953; Mitter et al. 1991; Schluter 2000; Yoder et al. 2010).

Host‐associated speciation in typically monophagous groups has generally been proposed to involve dietary expansion to include a novel host, followed by some degree of genetic divergence, host preference behavior, reproductive isolation, and adaptation to the novel host and its associated ecological features (Diehl and Bush 1984; Jaenike 1990; Schluter 2000; Berlocher and Feder 2002; Coyne and Orr 2004; Rundle and Nosil 2005). The extent to which the initial divergence is facilitated by adaptation via differential natural selection on the different host species is unknown. The role of adaptation to different habitats or environments (e.g., host plants for phytophagous insects) in speciation has been termed “ecological speciation” and may be associated with diversification in both plants and animals (Schluter 2000; Rundle and Nosil 2005). Ecological speciation as a process requires that (1) natural selection is the primary cause of divergence and (2) this divergence leads to, or is associated with, reproductive isolation (Schluter 2001).

Ecological speciation is a useful, if not universal, framework to explain high rates of diversification in situ, without having to postulate multiple occurrences of allopatric separation (Coyne and Orr 2004). Under a scenario of ecological speciation, natural selection alone can result in genetic divergence, not only of those traits directly related to use of the novel environment but also in associated traits potentially contributing to reproductive isolation (Schluter 2009; Matsubayashi et al. 2010). Of course, additional information on behavior, genetics, phylogeography, and ecology is critical for providing context to the core ecological speciation hypothesis for any particular set of populations.

To determine whether ecological speciation may contribute to any particular radiation of phytophagous insects on host plants, it is necessary to investigate how genetics and ecology interact in currently diverging or recently evolved taxa within the radiation. Because diverging populations are typically too similar for us to differentiate morphologically, the initial sign of host‐associated divergence may be a behavioral, phenological, or other ecological shift. In the past, such observations could only be explored by experimental studies on host‐associated preference, oviposition, and performance traits. Recently, an arsenal of molecular laboratory markers and analyses are available for assessing genetic indicators of divergence and evaluating hypotheses about the evolutionary mechanisms at play (Storz 2005; Excoffier and Heckel 2006; Ekblom and Galindo 2011). In particular, measures of genetic structure, level(s) of divergence, gene flow, natural selection, phylogeography, and directionality involved with a host shift are most useful for understanding the magnitude and the underlying processes causing an ecologically mediated divergence (Rundle and Nosil 2005; Schluter 2009; Schluter and Conte 2009).

The agromyzid leafmining genus *Phytomyza* (Diptera: Agromyzidae) contains more than 600 species, with host‐associated radiations accounting for much of the diversity (Winkler et al. 2009a,b). *Phytomyza glabricola* Kulp, a species endemic to the eastern United States, belongs to a radiation of 14 closely related species, all of which feed, as juveniles, within leafmines on various species of hollies, the genus *Ilex* (Aquifoliaceae) (Kulp 1968; Scheffer and Wiegmann 2000; Lonsdale and Scheffer 2011). Unlike its generally monophagous congeners, *P. glabricola* feeds on two sister species of holly, *Ilex glabra* (L.) A. Gray and *Ilex coriacea* (Pursh) Chapm, both of which are endemic to the eastern United States (Selbach‐Schnadelbach et al. 2009; Manen et al. 2010). Adult flies reared from leafmines from each host do not differ morphologically in either external characters or genitalia (Scheffer 2002; Lonsdale and Scheffer 2011).

**Figure 1:**
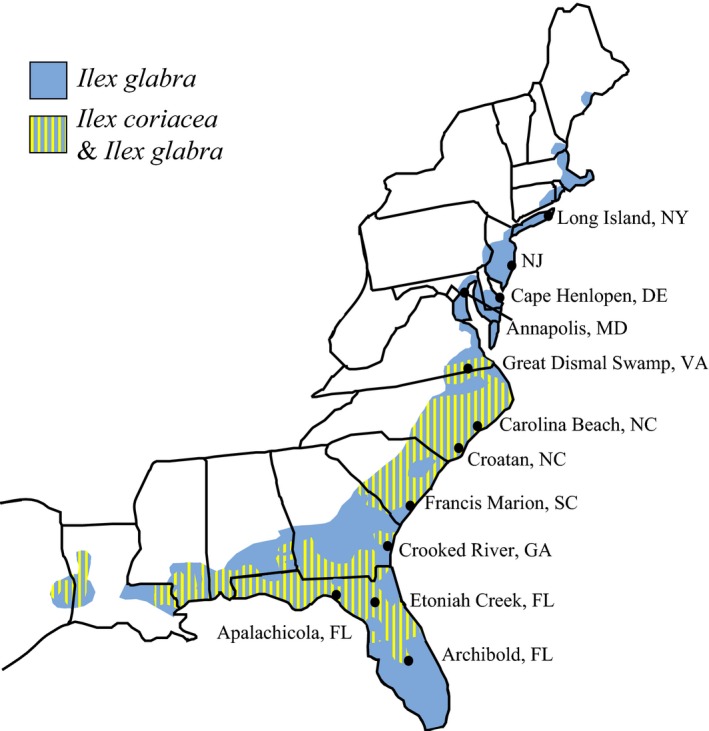
Endemic range of the host plants, *Ilex coriacea* and *I. glabra* with collection sites labeled.

*Ilex glabra* and *I. coriacea* are found in the coastal plains of the eastern United States (Caughey 1945; Richardson 1983, 1991; Brooks et al. 1993). *Ilex glabra* is present from Maine to southern Florida and west to northeastern Texas (Fig. 1). *Ilex coriacea* is sympatric with *I. glabra,* but it has a much smaller distribution, limited to the southern portion of *I. glabra's* range from North Carolina to northern Florida to Texas (Scheffer 2002). Where sympatric, the plants are often also syntopic, with leaves from one plant commonly in contact with leaves of the other species. Both phenotypic and genetic evidence from these two plant species indicates some degree of hybridization between the two host species in nature (Hebert , Scheffer, and Hawthorne unpubl. ms.).

In the field, when feeding on *I. glabra, P. glabricola* (hereafter “glabra‐flies”) have a development time of approximately 2–4 weeks with several generations a year. *Phytomyza glabricola* feeding on *I. coriacea* (“coriacea‐flies”) have a development time of 9–10 months with a single generation a year (Kulp 1968; Al‐Siyabi and Shetlar 1998; Scheffer 2002; Scheffer and Hawthorne 2007). Despite these phenological differences, adult *P. glabricola* from both hosts emerge in synchrony once a year in mid‐January to mid‐February, potentially allowing mating of flies from the two host plant species (Scheffer 2002), although laboratory mating experiments produced no adult fly hybrid offspring (Hebert et al. 2013).

Previous work revealed that *P. glabricola* populations from North and South Carolina show host plant‐based genetic divergence based on amplified fragment length polymorphism (AFLP) frequencies (Scheffer and Hawthorne 2007). However, we do not know whether the observed pattern of AFLP divergences exists throughout the range of these leaf miners. Such patterns could differ given the broad latitudinal span encompassing a variety of ecological conditions, including photoperiodism, temperature, plant quality, and natural enemy communities.

The goals of this study were threefold: (1) to explore the phylogeographic structure of host‐associated genetic variation across the ranges of *P. glabricola* and its host plants, (2) to investigate the role of natural selection associated with genetic divergence exhibited by host‐associated populations of these flies, and (3) to determine the directionality of the host shift or dietary expansion of *P. glabricola* involving the two host plants. To accomplish these goals, we collected DNA sequence data from the nuclear protein‐coding gene elongation factor‐1*α* (EF‐1*α*) and genomewide AFLP data from *P. glabricola* reared from *I. glabra* and *I. coriacea* throughout its range.

## Materials and Methods

### Collections

We greatly expanded the geographic sampling of *P. glabricola* from two locations in the Carolinas (Scheffer and Hawthorne 2007) to 11 locations from New York to Florida, essentially spanning most of the geographic range of *I. glabra* and all of the range of *I. coriacea*. Flies were collected in January and February of 2006 from Croatan National Forest in North Carolina and Francis Marion National Forest in South Carolina, and again in 2007 with additional samples from Cape Henlopen State Park, DE; the Great Dismal Swamp National Wildlife Refuge, VA; Crooked River State Park, GA; Etoniah Creek State Forest, FL; and Apalachicola National Forest, FL (Fig. 1). *Ilex glabra* was found at every collection site; however, *I. coriacea* was absent from the most northern sites (NY, NJ, DE, MD), which are outside the plant's geographic range, and from the GA and Archbold, FL sites. Leaves containing well‐developed leafmines, and visible larvae, were removed from host plants and placed into plastic bags labeled for site and host plant species. Pupae were dissected from mines and placed individually in 0.5‐mL Eppendorf tubes and stored in a moist chamber until adults emerged. Adult flies were placed in 100% ethanol and stored at −80°C.

### AFLPs

Genomic DNA was extracted from 183 individual flies (96 coriacea‐flies and 87 glabra‐flies) following the animal tissue protocol of the Qiagen DNeasy kit (Qiagen, Valencia, CA). DNA concentrations were standardized to 12.5 ng/*μ*L. AFLP constructs, preamplification, and amplification were performed as in Murray and Hare (2006) with minor modifications using the primers found in Table S1. PCR products were separated with an ABI 3730 DNA Analyzer (Applied Biosystems, Carlsbad, CA) using MapMarker X‐Rhodamine (ROX) labeled 1000‐bp ladder (BioVentures, Murfreesboro, TN).

Electropherograms were scored using genemapper v.3.7 (Applied Biosystems, Carlsbad, CA). Fragments between 76 and 800 base pairs were first scored using the automated procedure and secondarily checked by eye. To measure the repeatability of peaks, six individuals were repeated across plates and an additional ten individuals replicated within each plate. Negative controls (H_2_O template) were included at every step of the process. A genotyping error rate was estimated as the ratio of electropherogram peak mismatches among the replicates to the total number of replicated markers (Pompanon et al. 2005). Loci with peak mismatches among repeated samples were removed from the analysis as were loci occurring at the same sizes as peaks observed in the negative controls. Mismatches were not equally distributed among loci: Some loci had only a single individual with a mismatch, whereas others showed mismatches in a large number of individuals. Therefore, the percentage of loci removed due to mismatches was much higher than the overall genotyping error rate. Finally, because a significant negative correlation of fragment frequency and fragment size may be caused by excessive homoplasy, we estimated that correlation using AFLpsurv (Vekemans et al. 2002).

Estimates of allele frequencies and LD between AFLP markers were performed as described by Hill (1974) using the statistical package R (v2.11.1, 2010; Appendix A; code available upon request). A chi‐square test with one degree of freedom was used as an approximation of the likelihood ratio of LD to no LD to infer significance of LD comparisons (Hill 1974). We used a correction for multiple comparisons based on false discovery rates (Pike 2011) for all tests of LD to account for multiple nonindependent comparisons.

### Nuclear sequence data

Elongation factor‐1*α* (EF‐1*α*) is a fairly conserved nuclear protein gene generally used for higher‐level phylogenetics above the genus level (Mitchell et al. 1997; Shultz and Regier 2000; Scheffer et al. 2007). Preliminary data exploring EF‐1*α* for a phylogenetic analysis of Agromyzidae found a single base pair difference between *P. glabricola* from *I. glabra* and *I. coriacea* (S. J. Scheffer unpubl. data), which we chose to explore further in this study. A 910‐bp DNA fragment of the nuclear protein‐coding gene was amplified from genomic DNA of 236 flies (122 coriacea‐flies and 114 glabra‐flies) collected in 2006 and 2007, and 46 flies (25 coriacea‐flies and 21 glabra‐flies) from a previous study (Scheffer and Hawthorne 2007) using the primers found in Table S1. A standard amplification protocol was used to amplify the fragments, with initial denaturation at 95°C for 2 min followed by 12 cycles of 92°C for 15 sec, 56°C for 30 sec, and 70°C for 1 min 30 sec, then 32 cycles of 92 °C for 10 sec, 55 °C for 15 sec, and 72 °C for 1 min 30 sec, with a final extension at 72 °C for 10 min. PCR products were purified using either the QIAquick PCR purification kit or the QIAquick gel extraction kit (Qiagen, Inc.). Purified PCR product was used in sequencing reactions with BigDye sequencing kits (Applied Biosystems, Foster City, CA) and the products generated using an ABI‐3130 automated sequencer (Applied Biosystems). Diploid sequencing was conducted using nested primers to ensure overlap of at least two amplifications for each sample (Table S1). Sequence contigs were assembled and aligned using codoncode aligner (v.2.0 CodonCode Corp., Dedham, MA). Heterozygous states were identified as dual peaks. The reading frame of the final consensus sequence was determined by comparison with EF‐1*α*100E and EF‐1*α*48D in *Drosophila melanogaster*.

Allelic phase for EF‐1*α* sequences was reconstructed using the program cvhaplot (v.2.01 Huang et al. 2008; Huang and Zhang 2010). cvhaplot runs the sequences through several phase‐determining programs, each of which has a different algorithm for phase determination, and the resulting haplotypes are compared among analyses to check for consensus between programs. cvhaplot was run with the entire dataset, then with flies from each host plant separately. Results from separate analyses of the flies by hosts were more consistent among phase‐determining programs; therefore, they were used to determine phase. Taking a conservative approach, only samples with agreement in five or more programs were included in subsequent analyses (Huang et al. 2008; Huang and Zhang 2010). Sequences from each individual have been submitted to GenBank under the accession nos. JX658138–JX658435.

### Geography of host plant‐associated genetic divergence

Host plant‐associated genetic differentiation was estimated for the entire dataset as well as within geographic locations using both AFLP markers and EF‐1*α* sequence data. For AFLPs, 5000 permutations were run to calculate and test the significance of *F*_ST_ using aflpsurv (v.1.0 Vekemans et al. 2002). For EF‐1*α* sequence data, Φ_ST_ (Excoffier et al. 1992) was estimated using arlequin (v.3.5 Excoffier et al. 2005). *F*_ST_ and Φ_ST_ for within‐host comparisons among locations were calculated only if at least five individuals were present in a population on a single host plant species; for among‐host comparisons, locations were only included if at least five individuals were present on each host plant species.

We took two additional approaches to measuring the genetic divergence among flies collected from different plant species and locations: An analysis of molecular variance was performed using a permutational MANOVA via the adonis function from the vegan package (Oksanen et al. 2010) in the statistical package R (v 2.11.1, 2010), and using a clustering method that required no a priori hypotheses of substructure using the AFLP data. The distance matrix for EF‐1*α* was calculated using the F84 model of nucleotide substitution (Kishino and Hasegawa 1989; Felsenstein and Churchill 1996) in dnadist, part of the phylip package (v.3.69 Felsenstein 2005); Jaccard distances were calculated using the AFLPs because they are based only on the shared presence of peaks. adonis models were constructed to test the effects of host plant source and collection site location on the genetic structure of flies from all locations, and effects of collection year using only the locations common to all years collected. The mean allele frequencies did not differ between sample years for AFLPs (*F* = 1.17435, df = 1, *P* = 0.226, Table S2) nor EF‐1*α* (*F* = 2.2811, df = 1, *P* = 0.168, Table S3); therefore, the datasets from the 2 years were combined. adonis models were also constructed to test the effects of sex of the fly on genetic structure of the flies using AFLP data. Models were run with host plant source nested within location. Significance was based on 5000 permutations producing pseudo‐F ratios.

Second, we performed nonmetric multidimensional scaling (NMDS) on pairwise Jaccard genetic distance estimates between individual genotypes to visualize the data in two dimensions. Using NMDS, we are also able to estimate the correlation of a series of explanatory variables, including host plant source, sex, and year, with genetic distances among individuals. The ordination was generated using the function metamds, also part of the vegan package in R, and the magnitudes of variance attributable to the categorical explanatory variables were tested using a goodness‐of‐fit statistic based on 5000 permutations of environmental variables on the ordination data using the function envfit in R.

### Host‐associated divergent selection

We used genome scans to identify AFLP loci whose divergence exceeded that expected by neutral processes in order to infer the action of selection in causing genetic divergence among flies using the two host plants. We used two methods to detect outliers in several geographic locations to gain confidence in our results by rejecting false positives that are identified in single comparisons and with different analyses (Luikart et al. 2003; Stinchcombe and Hoekstra 2008). First, we identified AFLP loci with exceptionally divergent allele frequencies using a hierarchical Bayesian approach in dfdist (Beaumont and Balding 2004), and then, we directly asked which loci may have diverged by selection using bayescan (v.1.0 Foll and Gaggiotti 2008). To generate a seed for creation of a null distribution of *F*_ST_ in dfdist, a trimmed mean *F*_ST_ for each population was estimated that excludes the highest and lowest 30% of locus‐specific *F*_ST_ estimates in the AFLP dataset (Weir and Cockerham 1984; Zhivotovsky 1999; Bonin et al. 2006). Loci with an *F*_ST_ in the upper 95% and 99% confidence intervals of the simulated distributions were labeled “outliers” and are candidates for divergent selection. dfdist was run using the total dataset, then separately for populations with greater than five individuals on each host (NC, SC, and eastern Florida), as loci repeatedly identified in more than one location are considered especially robust (Campbell and Bernatchez 2004; Bonin et al. 2006; Egan et al. 2008; Nosil et al. 2008; Hohenlohe et al. 2010).

Unlike dfdist, bayescan estimates the posterior probability of a given locus under two models, evolving neutrally or under selection, using a reversible MCMC approach in which *F*_IS_ is allowed to vary between zero and one. bayescan was run starting with a burn‐in period of 20 pilot runs, each with a length of 10^4^ iterations. The burn‐in was followed by 40 thinning intervals each with 10^4^ iterations for a total of 400,000 iterations. Outliers were identified as loci with posterior probabilities of being under selection at the “strong” (0.91–0.97), “very strong” (0.97–0.99), and “decisive” (>0.99) levels.

### Identification of ancestral and novel host plants

To determine the direction of the initial host range expansion, we estimated diversity and genetic structure using the EF‐1*α* dataset. We expected more genetic variation and older haplotypes in flies from the ancestral host plant (Harrison 1991; Brown et al. 1996), whereas there should be no difference in the diversity and relative age of haplotypes if both host forms of flies arose at the same time or if the divergence is very old.

We compared the diversity of EF‐1*α* haplotypes of flies from the two host plant species. For EF‐1*α* sequence data, the number of haplotypes (H), polymorphic sites (p), haplotype diversity (Hd, Nei 1987), and nucleotide diversity (*π*, Tajima 1983) was estimated using arlequin (v.3.5 Excoffier et al. 2005). The number of singleton haplotypes (S_n_) and nucleotide diversity (*π*, Tajima 1983) was estimated using and dnasp (v.5.1 Librado and Rozas 2009). To visualize the relationships among EF‐1*α* haplotypes found in flies from each host plant, we generated a median‐joining network using network (v.4.5.1.6 Bandelt et al. 1999; Polzin and Daneschmand 2003). The network was rooted using EF‐1*α* sequences from three closely related species: *P. ilicis*,* P. ilicicola*, and *P. ditmani* to further inform our inference of the relative ages of haplotypes from different host plants (Winkler et al. 2009b).

Levels of genetic diversity were also compared across the two hosts using the AFLP dataset. Nei's genetic diversity (H_J_) and the average gene diversity within populations (H_S_) were calculated using aflpsurv (v.1.0 Vekemans et al. 2002).

## Results

A total of 656 AFLP markers were scored from 183 flies with an initial error rate of 5.5% (Table 1). We removed 258 markers due to discrepancies across repeated samples. No plate effect was found. Linkage disequilibrium analyses resulted in patterns of linked markers of the same size from different primer pairs, indicating nonspecific primer binding. All but one linked locus was discarded to eliminate replicated markers, resulting in a total of 305 markers. Finally, any markers where only one individual contained the rarer allele were discarded, giving a final total of 265 markers. The size range of the AFLP markers was 78–792 bp, and 92% were longer than 200 bp. The Pearson correlation coefficient between fragment sizes and fragment frequencies was not significant (*r* = −0.0137, *P* = 0.82310), indicating a low risk of homoplasy due to small fragment sizes (Vekemans et al. 2002).

**Table 1:** Summary of samples genotyped from each location and year

State	Site	Population	Coriacea‐flies					Glabra‐flies				
			EF‐1*α*			AFLP		EF‐1*α*			AFLP	
			S&H^a^	2006	2007	2006	2007	S&H^a^	2006	2007	2006	2007
FL	Apalachicola National Forest	Hunters	–b	–	6	–	6	–	–	1	–	1
	Archbold Biological Station	Archibald	–	–	–	–	–	8	–	–	–	–
	Etoniah Creek State Forest	East V	–	–	–	–	–	–	–	2	–	0
		Stuck in Sand	–	–	7	–	5	–	–	11	–	9
GA	Crooked River State Park	Crooked River	–	–	–	–	–	–	–	4	–	3
SC	Francis Marion National Forest	Big Ocean Bay	10	17	5	15	4	–	18	6	17	4
		Wambaw Trail	–	19	10	12	7	–	21	1	16	2
NC	Croatan National Forest	Catfish Lake	–	22	–	18	–	–	5	–	3	–
		Road 152	–	22	10	20	7	–	25	4	19	2
	Carolina Beach State Park	Carolina Beach	15	–	–	–	–	7	–	–	–	–
VA	Great Dismal SwampNational Wildlife Refuge	Great Dismal Swamp	–	–	4	–	2	–	–	1	–	1
MD	Annapolis	Annapolis	–	–	–	–	–	2	–	–	–	–
DE	Cape Henlopen State Park	Cape Henlopen	–	–	–	–	–	–	–	15	–	10
NY	Long Island	Long Island	–	–	–	–	–	4	–	–	–	–
		Subtotal	25	80	42	63	31	21	69	45	55	32
		Total	147			96		135			87	

a Details on samples can be found in Scheffer and Hawthorne (2007).

b Samples not collected from locations with “–”.

A total of 308 flies were genotyped for a 910‐bp sequence of EF‐1*α*, resulting in 27 SNPs (Table 1). Only 279 individuals (145 coriacea‐flies and 134 glabra‐flies) had five or more votes in the consensus analysis of cvhaplot, reducing the dataset to 75 distinct genotypes and 43 haplotypes with 22 polymorphic sites (Table S4). All polymorphic sites were in the third codon positions; however, five would be expected to result in amino acid changes, four with changes between asparagine and lysine, and one between aspartic acid and lysine.

### Geographic scale of host plant‐associated genetic divergence

Significant genetic divergence was found among host‐associated populations using both AFLPs (*F*_ST:_ 0.1247, *P* < 0.0005) and EF‐1*α* (Φ_ST:_ 0.50744, *P* < 0.001; Table 2). Results from the MANOVA gave the same pattern (Tables S2 and S3). Host‐associated genetic differences were also significant within North Carolina, South Carolina, and eastern Florida, although the magnitude of divergence varied among locations (Table 2). There were significant, but smaller, differences in allele frequencies among flies within the same host plant species from different locations, both for AFLPs (coriacea‐flies: *F*_ST_: 0.0482, *P* = 0.0182 glabra‐flies: *F*_ST_: 0.0178, *P* = 0.0146; Table 2) and EF‐1*α* (coriacea‐flies: Φ_ST_: 0.02844, *P* = 0.00880; glabra‐flies: Φ_ST_: 0.01263, *P* = 0.09677; Table 2).

**Table 2:** Estimates of *F*_ST_ from AFLPs and EF‐1*α* based on host plant (total samples), host plant within locations, and among locations within coriacea‐flies and glabra‐flies (separately). Samples from locations with less than five samples on one of the host plants were removed from all but the host plant comparison

Comparison	AFLPs		EF‐1*α*		AFLP outliers		AFLP nonoutliers	
	*F*_ST_	*P*‐value	Φ_ST_	*P*‐value	*F*_ST_	*P*‐value	*F*_ST_	*P*‐value
Host plant	0.1247	<0.0005	0.5166	<0.0001	0.4946	<0.0005	0.0571	<0.0005
NC host plant	0.1270	<0.0005	0.4950	<0.0001	0.5045	<0.0005	0.0632	<0.0005
SC host plant	0.1390	<0.0005	0.5599	<0.0001	0.5179	<0.0005	0.0764	<0.0005
East‐FL host plant	0.0973	0.0032	0.5892	<0.0001	0.4154	<0.0005	0.0553	0.0142
Locations of coriacea‐flies	0.0482	0.0182	0.0284	0.0088	0.0883	0.0076	0.0312	0.0014
Locations of glabra‐flies	0.0178	0.0146	0.0374	0.0968	0.0527	0.0834	0.0158	0.0322

**Figure 2:**
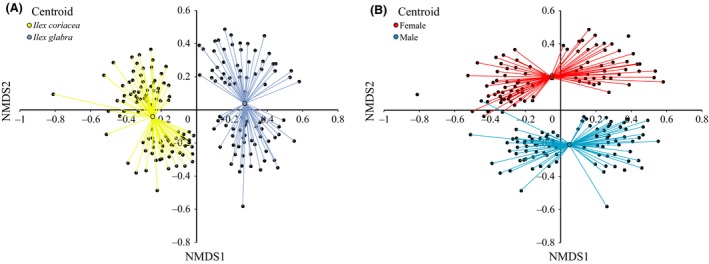
Results of nonmetric multidimensional scaling (NMDS) of AFLPs. (A) Yellow represents flies from *Ilex coriacea,* and blue represents flies collected from *I. glabra*. B) Red represents female flies and blue represents male flies. Four samples were genotyped as larvae; therefore, their sex is unknown.

Using the AFLP data, the 183 individuals formed four distinct groups on the first two NMDS axes, corresponding to host plant and sex of the fly (Fig. 2). The Kruskal's stress for the final ordination was 22.9%. Both host plant and sex were significantly correlated with the ordination of the AFLP data (host plant: *R* ^2^ = 0.4965, *P* = 0.0002, Fig. 2; sex: *R* ^2^ = 0.3123, *P* = 0.0002, Fig. 2). Visually, flies from each host plant clearly separated along the first axis, and males and females separated along the second axis (Fig. 2). The differentiation among the sex of the flies indicates good coverage of the genome.

### Host‐associated divergent selection

**Figure 3:**
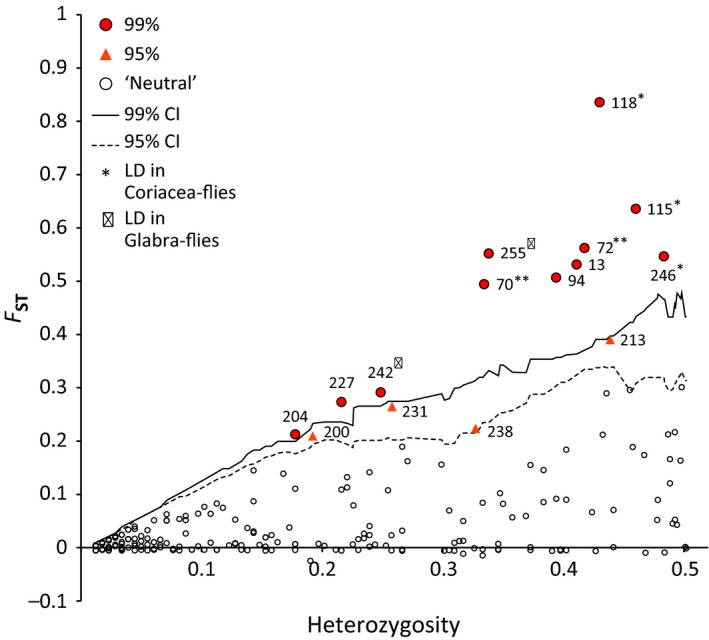
Outlier analysis from among host plant comparison of AFLP data from *Phytomyza glabricola* using dfdist. Lines represent the 95% and 99% confidence intervals generated from the trimmed mean *F*_ST_ in dfdist.

Migration, mutation, drift, and inbreeding are expected to affect all loci in a genome in a similar fashion. In contrast, selection should have locus‐specific effects: Selected sites should show lower genetic diversity and increased genetic differentiation among populations with contrasting environments relative to the rest of the genome (Beaumont and Balding 2004; Egan et al. 2008; Nosil et al. 2009). Genome scans in dfdist testing for divergent selection between populations on each host plant in each location indicated 32 loci (12.5%) with *F*_ST_ higher than the 95th percentile of the simulation results in at least one comparison (Table S5). Of those, 24 (9.3%) were significant outliers in multiple locations. When all populations were combined, 15 (5.7%) outliers were significant among host plants (Fig. 3; Tables S5 and S6). All but two (loci 200 and 238) of the 15 loci found in the combined comparison were also significant using bayescan (Table S6), and all but locus 238 were significant in multiple independent comparisons. These two loci were the closest outliers to the 95% cutoff in dfdist, so they had a lower likelihood in general of being outliers (Fig. 3).

The values of Φ_ST_ in EF‐1*α* were very similar to the *F*_ST_ estimates using only outlier AFLP loci (Table 2). Given the high F_ST_, but the lack of nonsynonymous changes in the DNA sequence, the data suggest EF‐1*α* is likely near a locus under divergent selection. When EF‐1*α* is added to the AFLP outliers present in multiple independent comparisons, there appear to be 15 loci showing signs of divergent selection among host plants in these flies.

Genome scans among samples of coriacea‐flies collected from different locations identified 13 loci (6.4%) as significant outliers in dfdist (Tables S5 and S6). None of those markers were outliers in more than one independent comparison, and only one was identified in bayescan (locus 144 within coriacea‐flies; Table S6). Two of the 13 outlier loci were also identified as host‐associated outliers (locus 70 and locus 72; Table S6). Upon further examination, the outlier status appeared to be driven by coriacea‐flies in eastern Florida (Table S7). These populations lack a fixed absence in locus 70 and a nearly fixed presence in locus 72 found in all other populations of flies collected from *I. coriacea* (Table S7).

Genome scans of glabra‐flies found fewer location‐specific outliers: Ten loci (5.1%) were identified at the 95% level in dfdist (Tables S5 and S6), none of which were significant in bayescan. Two of the among‐location outliers identified in glabra‐flies were also outliers in coriacea‐flies in dfdist, but did not exhibit corresponding geographic patterns (locus 167 and locus 226; Table S6). Location‐associated divergence for locus 167 was driven by genetic differences in eastern Florida for both coriacea‐flies and glabra‐flies (Table S7). Locus 226 was more complicated: Among coriacea‐flies, differences existed between both northern populations and Florida, and between eastern and western Florida populations; among glabra‐flies, differences were driven by the population in Delaware (Table S7).

### Identification of ancestral and novel host plants

**Figure 4:**
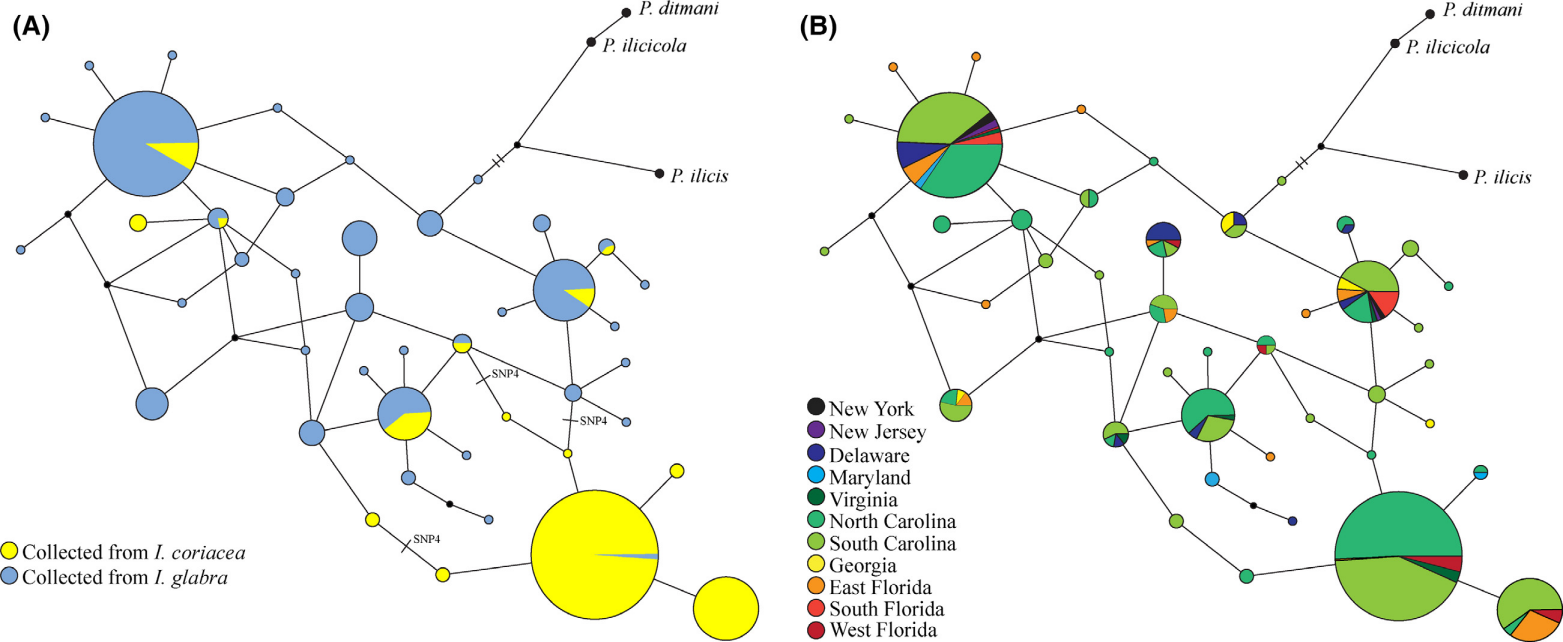
Haplotype network of EF‐1*α* in *P. glabricola*. The size of nodes reflects the relative abundance of each haplotype in the total population. Each connection represents one base pair difference between nodes. The network is rooted by three closely related species: *P. ilicis, P. ditmani, and P. ilicicola*. (A) Nodes are colored based upon the frequency of flies from each host plant with that haplotype. (B) Nodes are colored based upon the geographic location from which each fly was collected.

The mean genetic variability of EF‐1*α* was consistently less among coriacea‐flies than glabra‐flies regardless of the measure used (Table 3). Coriacea‐flies have less haplotype and nucleotide diversity and fewer average pairwise differences than glabra‐flies. In addition, there were 14 haplotypes and only one singleton found in coriacea‐flies, whereas glabra‐flies had more than twice as many haplotypes (36) and 13 singletons (Fig. 4A). There were seven haplotypes shared by flies from the different host plants. The most common haplotype (h13) was found in 126 of the 135 coriacea‐flies but only one glabra‐fly (Fig. 4A). In contrast to host‐associated structure observed in the haplotype network, no geographic patterns were present in the network (Fig. 4B).

**Table 3:** Summary statistics for EF‐1*α* sequence data

	*N*	*H*	*p*	Sn	*H*_d_	Π	Rm
Flies from *I. coriacea*	145	15	10	1	0.4932	0.047474	4
Flies from *I. glabra*	134	36	22	13	0.7869	0.104536	5
Total flies	279	43	22	12	0.8008	0.114040	6

*N*, number of phased samples; *H*, the number of haplotypes; *p*, the total number of polymorphic SNPs; Sn, the number of singleton haplotypes; *H*_d_, haplotype diversity; *π*, nucleotide diversity; Rm, the minimum number of recombination events.

The majority of the EF‐1*α* haplotypes found in coriacea‐flies formed a cluster distal to the most similar haplotypes found in glabra‐flies. This cluster is distinguished by alternative alleles of a single SNP (snp4; Fig. 4A). The SNP was nearly a fixed difference between host forms; however, 2.8% of coriacea‐flies are homozygous for the glabra‐fly allele, 19.3% of coriacea‐flies were heterozygous, and 0.7% of glabra‐flies were homozygous for the coriacea‐fly allele. No glabra‐flies were heterozygous at this position.

In contrast to EF‐1*α* data, estimates of genetic diversity in the AFLP dataset were fairly similar among flies from the two host species. Glabra‐flies had slightly higher values for Nei's genetic diversity (coriacea‐flies: 0.1559, glabra‐flies: 0.1594) and Nei's H_S_ (coriacea‐flies: 0.1723, glabra‐flies: 0.1771), but coriacea‐flies had slightly more polymorphic loci than glabra‐flies (coriacea‐flies: 238, glabra‐flies: 232; Table 4). The results were similar with nonoutlier loci (Table 4); however, glabra‐flies have two more polymorphic outlier loci than coriacea‐flies (14 vs. 12, Table 4).

**Table 4:** Summary statistics for AFLPs: (a) all loci combined, (b) outlier loci only, (c) nonoutlier loci only

Pop	*n*	#loc.	#poly. loc.	*H*_J_	*H*_S_
(a)					
Flies from *I. coriacea*	96	265	238	0.1559	0.1723
Flies from *I. glabra*	87	265	232	0.1594	0.1771
Total	183	265	265	0.1662	0.1577
(b)					
Flies from *I. coriacea*	96	15	12	0.2429	0.2404
Flies from *I. glabra*	87	15	14	0.2594	0.2444
Total	183	15	15	0.3430	0.2512
(c)					
Flies from *I. coriacea*	96	250	226	0.1508	0.1590
Flies from *I. glabra*	87	250	218	0.1535	0.1646
Total	183	250	250	0.1556	0.1521

*n*, number of samples; #loc., number of loci; #poly loci., number of polymorphic loci; *H*_J_, Nei's gene diversity; *H*_S_, average gene diversity within populations.

## Discussion

Under a scenario of ecological speciation, natural selection alone can result in genetic divergence and correlated/associated traits ultimately resulting in reproductive isolation (Schluter 2009; Matsubayashi et al. 2010). We investigated the questions of whether host‐associated populations of *P. glabricola* are genetically diverged and whether they exhibit genetic evidence of natural selection throughout their ranges. In addition, we attempt to infer the direction of a putative host shift that might have been associated with the divergence.

### Geographic scale of divergence

We found strong evidence of host‐associated genetic divergence across the range of *P. glabricola*, supporting the previous determination, based on limited geographic sampling, of coriacea‐flies and glabra‐flies as host‐associated forms (Scheffer and Hawthorne 2007; Funk 2012). With our increased geographic sampling, we have found that divergence among flies from different host plants is consistently much larger than divergence among different locations within a given host. For example, coriacea‐flies from Florida are genetically more similar to coriacea‐flies from Delaware than they are to glabra‐flies from Florida (Table 2). Although genetic divergence between glabra‐flies and coriacea‐flies is present at all locations, the degree of that divergence between host‐associated sympatric populations varies slightly by location. Thus, there was no clear geographic pattern to the variation in genetic divergence among locations. It is possible that the geographic variation in estimates of F_ST_ is a result of variation in sample sizes taken from the different locations.

### Host‐associated divergent selection

Genome scans identified 13 AFLP loci with a higher *F*_ST_ between host‐associated populations than expected due to genetic drift alone. These outliers represent loci with frequencies more different between host‐associated populations than expected under neutral evolution by genetic drift and most likely represent regions of the genomes experiencing host‐associated natural selection. No AFLP outlier loci were found, via the criteria used here, among populations of flies sampled from the same host plant, indicating that divergent selection is much stronger between host plants than is geographically mediated local adaptation (Table S6).

Sequence data at EF‐1*α* also showed differences in Φ_ST_ among locations. Estimates of Φ_ST_ were similar in magnitude to the significant outliers in the genome scan of AFLPs. This level of divergence suggests that EF‐1*α* is either a target of selection directly, or more likely, near a locus under divergent selection. We favor an explanation of physical or functional linkage of the selected gene to EF‐1 to loci that are the direct targets of selection because although several of the observed polymorphisms are expected to result in amino acid substitution, we are unaware of previous observations that this gene underlies variation in host plant use or seasonality in insects.

Aside from hybridization behavior or success, the only phenotypic character known to be associated with the divergence in *P. glabricola* on the two host species is the difference in life history. Glabra‐flies have multiple generations each year (multivoltine), while coriacea‐flies have an unusually long larval development period involving only a single generation each year (univoltine). The univoltine life cycle is unusual in agromyzid flies, being known only in several other species of *Phytomyza* holly leaf miners feeding on evergreen hollies. In the case of *P. glabricola* on the two host plants, the phonological difference in development does not translate into phenological isolation of adult emergence; once a year, in the spring, flies from the two hosts emerge synchronously and have the potential to mate. Interestingly, this results in every generation of coriacea‐flies experiencing the presence of glabra‐flies, but only ~one in four generations of glabra‐flies seeing coriacea‐flies. In any case, the difference in generation time appears to be an important trait with the potential for impact on evolutionary processes. However, it remains unclear as to whether this difference is a cause or consequence of the divergence in *P. glabricola*.

### Direction of host range expansion

The topology of the EF‐1*α* haplotype network suggests that *I. glabra* may be the ancestral host of *P. glabricola* with a recent shift to *I. coriacea*. Haplotypes carried by the glabra‐flies are more diverse and many occur internally in the network and therefore likely to be older than the coriacea‐fly haplotypes located primarily at the tips of the network (Donnelly and Tavare 1986; Golding 1987; Crandall and Templeton 1993). Furthermore, the haplotypes most characteristic of coriacea‐flies were found clustered as an offshoot of the main network. Finally, closely related holly leaf miner species used as outgroups for the network were most closely related to glabra‐fly haplotypes (Fig. 4A).

This EF‐1*α* topology suggests that a subset of flies from *I. glabra* colonized *I. coriacea*, bringing along only a fraction of the ancestral allelic diversity, as would be expected (Harrison 1991; Brown et al. 1996). Subsequently, haplotypes within *I. coriacea* have arisen and, due to reduced gene flow with glabra‐flies, have remained restricted to the coriacea‐flies. An alternative scenario is that reduced EF‐1*α* haplotype variation in flies on *I. coriacea* is due to natural selection eliminating certain haplotypes of either EF‐1*α*, or a locus closely linked to it, in coriacea‐flies but not glabra‐flies. This could also be indicative of a host expansion or shift from *I. glabra* to *I. coriacea* if the effects of selection on allelic diversity are stronger on the novel host as might be expected, particularly in light of the dramatic lifestyle/feeding difference on the two hosts.

Although coriacea‐flies exhibited less genetic variation in EF‐1*α* than in glabra‐flies, the AFLP data indicated roughly equal genomic variation within the two host‐associated groups. If the reduced variation in EF‐1*α* among coriacea‐flies was due to a founder event and demographic bottleneck following a host range expansion, we would expect the AFLPs to show similarly reduced diversity in coriacea‐flies, as well, as drift should affect all loci similarly (Cavalli‐Sforza 1966; Lewontin and Krakauer 1973; Vitalis et al. 2001). As EF‐1*α* appears to be linked to a locus experiencing strong selection, reduction in variation in EF‐1*α* and linked loci could be occurring among coriacea‐flies without having genomewide effects on diversity throughout the genome, depending on the size of associated hitchhiking regions (Andolfatto 2001).

## Conclusions

*Phytomyza glabricola* leaf miners feeding on *Ilex coriacea* and *I. glabra* exhibit several characteristics consistent with what is expected from recent or ongoing ecological speciation. Flies sampled from *I. glabra* and *I. coriacea* are genetically diverged throughout their ranges, extending previous observations (Scheffer and Hawthorne 2007). We also show that a number of nuclear markers are diverged significantly more than would be expected from random forces such as genetic drift, suggesting that natural selection is driving divergence in these flies and that this divergence is reflected in the genotype frequencies measured here. We have previously demonstrated that hybridization of flies from the respective host plants has a 100% failure rate in the laboratory, indicating some measure of reproductive isolation that may carry into the field (Hebert et al. 2013). However, no host‐associated divergence in mitochondrial sequences has been found (Scheffer and Hawthorne 2007), suggesting that the nuclear sequence and AFLP divergence are recent or that some gene flow may occur in the field.

Several important aspects of host‐associated divergence in *P. glabricola* remain to be investigated. Patterns of host plant hybridization in the field could dramatically affect host use by the flies. Additional study of reproductive behavior of the flies, and particularly potential hybridization, is warranted. Ecological variables, such as parasitoid wasps, could be an important source of host plant‐associated natural selection on the leaf miners. Finally, the extended larval development period of the leaf miners on *I. coriacea* is very unusual among leafmining agromyzids and is a strikingly different from the more typical life cycle of the glabra‐flies. It remains to be seen whether this difference in life cycle is a driver or a consequence of the divergence and how it may be related to host‐associated adaptation and reproductive isolation.

## Data Accessibility

EF‐1*α* sequences archived at GenBank under the accession nos. JX658138–JX658435.

## Conflict of Interest

None declared.

## Supporting information


**Table S1.** AFLP and EF‐1*α* primer sequences.
**Table S2.** Analysis of molecular variance estimated using the ADONIS function for AFLP data from *Phytomyza glabricola* feeding on either *Ilex coriacea* or *I. glabra*.
**Table S3.** Analysis of molecular variance estimated using the ADONIS function for EF‐1*α* sequences from *Phytomyza glabricola* feeding on either *Ilex coriacea* or *I. glabra*.
**Table S4.** Results from CVHAPLOT.
**Table S5.** Outliers detected using DFDIST from comparisons between all study populations.
**Table S6.** Summary of outlier loci found in host, sex, and geographic comparisons.
**Table S7.** Distribution of peaks in host‐associated outliers.
