# Supplementary material for: Evidence for ecological speciation via a host shift in the holly leaf miner, Phytomyza glabricola (Diptera: Agromyzidae)
Source: Ecol Evol. 2016 Aug 23;6(18):6565–77. doi: 10.1002/ece3.2358 (PMC5058528; doi:10.1002/ece3.2358)
Supplement: Supplementary file 1 — Table S1. AFLP and EF‐1α primer sequences. Table S2. Analysis of molecular variance estimated using the ADONIS function for AFLP data from Phytomyza glabricola feeding on either Ilex coriacea or I. glabra. Table S3. Analysis of molecular variance estimated using the ADONIS function for EF‐1α sequences from Phytomyza glabricola feeding on either Ilex coriacea or I. glabra. Table S4. Results from CVHAPLOT. Table S5. Outliers detected using DFDIST from comparisons between all study populations. Table S6. Summary of outlier loci found in host, sex, and geographic comparisons. Table S7. Distribution of peaks in host‐associated outliers. [file ECE3-6-6565-s001.pdf]

Supplemental Information:
Evidence for ecological speciation via a host shift in the holly leaf miner, *Phytomyza glabricola* (Diptera: Agromyzidae)

SUPPLEMENTAL TABLES

Table S1. AFLP and EF-1α primer sequences. *Pst1*A was used in combination with each of the *Eco*RI based primers (*E*ACA-*E*AGT).

Primer	Sequence
**AFLP**	
*Pst*1A	5 ’- GAC TGC GTA CAT GCA GA - 3’
*E*ACA	5’ - /56-FAM/GAC TGC GTA CCA ATT CAC A - 3’
*E*ACT	5’ - /56-FAM/GAC TGC GTA CCA ATT CAC T - 3’
*E*AGA	5’ - /56-FAM/GAC TGC GTA CCA ATT CAG A - 3’
*E*AGT	5’ - /56-FAM/GAC TGC GTA CCA ATT CAG T - 3’
**EF-1α**	
EF46F *	5' - GAG GAA ATC AAG AAG GAA G - 3'
PEF40F	5' - TCG TCA TTG GAC ACG TAG ATT CAG G - 3'
PEF61R	5' - GAT GGT TCC AAC ATG TTA TCA C - 3'
PEF64R	5' - CGA CAC ATA AAG GCT TGG ATG GCA CC - 3'
PEF65R	5' - GTC TCA TGT CAC GCA CAG CGA AAC GAC - 3'

*(Cho et al. 1995)

Table S2. Analysis of molecular variance estimated using the ADONIS function for AFLP data from *Phytomyza glabricola* feeding on either *Ilex coriacea* or *I. glabra*. Variation was partitioned **(a)** among individuals on each host plant species nested within each location, sex of the flies, and the collection year for North and South Carolina populations; **(b)** among individuals on each host nested within each location and sex of the flies; **(c & d)** among locations and sex of the flies within each host plant species. All non-significant interactions were removed from the analysis.

	Source	d.f.	SS	MS	F - model	R^2	P (>F)
**a)**	Location	1	0.33913	0.33913	2.38946	0.0126	< **0.001**
	Sex	2	2.72593	1.36296	9.60317	0.1009	< **0.001**
	Year	1	0.16667	0.16667	1.17435	0.0062	0.226
	Host nested in Location	2	4.05319	2.02659	14.27898	0.1500	< **0.001**
	Residuals	139	19.72807	0.14193	- .	0.7303	- .
	Total	145	27.01300			1	
**b)**	Location	6	2.30711	0.38452	2.71925	0.0670	< **0.001**
	Sex	2	3.23548	1.61774	11.44043	0.0939	< **0.001**
	Host nested in Location	5	5.00615	1.00123	7.08055	0.1453	< **0.001**
	Residuals	169	23.89756	0.14141	- .	0.6938	- .
	Total	182	34.44630			1	
**c)**	**Coriacea-flies**						
	Location	4	1.20764	0.30191	2.28281	0.0806	< **0.0005**
	Sex	2	2.00981	1.00491	7.59829	0.1341	< **0.0005**
	Residuals	89	11.77062	0.13225	- .	0.7853	- .
	Total	95	14.98807			1	
**d)**	**Glabra-flies**						
	Location	6	1.55257	0.25876	1.73281	0.1046	< **0.0005**
	Sex	2	1.63939	0.81969	5.48911	0.1105	< **0.0005**
	Residuals	78	11.64781	0.14933	- .	0.7849	- .
	Total	86	14.83977			1	

Table S3. Analysis of molecular variance estimated using the ADONIS function for EF-1α sequences from *Phytomyza glabricola* feeding on either *Ilex coriacea* or *I.glabra*. Variation was partitioned **(a)** among locations, year, and among individuals on each host plant nested within location for North and South Carolina populations (the only populations sampled in more than one year); **(b)** among locations and host plants nested within location; **(c & d)** among locations and sex of the flies within each host plant species. All non-significant interactions were removed from the analysis.

	Source	d.f.	SS	MS	F - model	R^2	P (>F)
**a)**	Location	1	-0.000003	-0.000003	- 3.9021	- 0.0046	1
	Year	1	0.000002	0.000002	2.2811	0.0027	0.1678
	Host nested in Location	2	0.000483	0.000242	314.17	0.7465	< **0.0005**
	Residuals	215	0.000165	0.000001	- .	0.2554	- .
	Total	219	0.000647			1	
**b)**	Location	9	1.381623	0.153514	10.597825	0.1378	< **0.0005**
	Host nested in Location	5	4.823749	0.964750	66.601577	0.4810	< **0.0005**
	Residuals	264	3.824143	0.014485	- .	0.3813	- .
	Total	278	10.029514			1	
**c)**	**Coriacea-flies**						
	Location	4	0.000012	0.000003	6.8100	0.1639	< **0.05**
	Residuals	139	0.000060	0.000000	- .	0.8361	- .
	Total	143	0.000072			1	
**d)**	**Glabra-flies**						
	Location	9	0.18877	0.020974	13.744	0.4974	< **0.05**
	Residuals	125	0.19076	0.001526	- .	0.5026	- .
	Total	134	0.37953			1	

Supplemental Table S4. Results from CVHaplot. Analyzing flies from each host plant separately yielded a better consensus between the programs.

CV category	H	S	I	II	III	Overall
Individuals (combined data)	127	139	13	9	8	296
Individuals (from *I. coriacea*)	90	53	2	2	1	148
Individuals (from *I. glabra*)	37	86	11	7	7	148
Number distinct genotypes	10	56	10	9	8	93
Total distinct haplotypes	10	33	14	16	14	57
Number of category-unique haplotypes*	10	25	8	7	7	57
Frequency (%) of category-unique haplotypes in total sample	83.9	11.5	2.2	1.2	1.2	100

Note: All rows following the separate host plant analyses refer to the combined data from those separate analyses. H: homozygous individuals; S: individuals where all programs fully supported the same haplotype; I – III: number of dissenting consensus votes received in each category (e.g., I means only one program had a different solution than the others); * Haplotypes newly observed in each category.

Table S5. Outliers detected using DFDIST from comparisons between all study populations. Dashes indicate the trimmed mean F_{ST} was too low a value to run DFDIST. 'Repeated across comparisons indicates' the number of loci with an outlier above 95% in more than one location comparison (number in independent comparisons).

			**Outlier loci: 95% (99%)**	
	**Geographic Distance (km)**	No. of polymorphic loci	Total	%
**Across hosts**				
C_{NC} vs. G_{DE}	430	187	11 (5)	5.9% (2.7%)
C_{NC} vs. G_{NC}	0	198	12 (8)	6.1% (4.0%)
C_{NC} vs. G_{SC}	312	214	13 (8)	6.1% (3.7%)
C_{NC} vs. $G_{E\text{-}FL}$	722	181	8 (2)	4.4% (1.1%)
C_{SC} vs. G_{DE}	752	177	10 (5)	5.6% (2.8%)
C_{SC} vs. G_{NC}	312	190	10 (8)	5.3% (4.2%)
C_{SC} vs. G_{SC}	0	215	13 (10)	6.0% (4.7%)
C_{SC} vs. $G_{E\text{-}FL}$	424	172	9 (3)	5.2% (1.7%)
$C_{E\text{-}FL}$ vs. G_{DE}	1175	115	3 (0)	2.6% (0%)
$C_{E\text{-}FL}$ vs. G_{NC}	722	143	5 (0)	3.5% (0%)
$C_{E\text{-}FL}$ vs. G_{SC}	424	170	14 (3)	8.2% (1.8%)
$C_{E\text{-}FL}$ vs. $G_{E\text{-}FL}$	0	104	6 (0)	5.8% (0%)
$C_{W\text{-}FL}$ vs. G_{DE}	1284	125	4 (0)	3.2% (0%)
$C_{W\text{-}FL}$ vs. G_{NC}	870	198	10 (4)	5.1% (2.0%)
$C_{W\text{-}FL}$ vs. G_{SC}	558	175	13 (7)	7.4% (4.0%)
$C_{W\text{-}FL}$ vs. $G_{E\text{-}FL}$	264	115	4 (3)	3.5% (2.6%)
**Combined**	na	257	15 (11)	5.7% (4.2%)
	**Repeated across comparisons**		23 (14)	8.7% (5.3%)
**Within *I. coriacea***				
C_{NC} vs. C_{SC}	312	190	--	--
C_{NC} vs. $C_{E\text{-}FL}$	722	163	8 (5)	4.9% (3.1%)
C_{NC} vs. $C_{W\text{-}FL}$	870	165	7 (2)	4.2% (1.2%)
C_{SC} vs. $C_{E\text{-}FL}$	424	154	6 (3)	3.9% (1.9%)
C_{SC} vs. $C_{W\text{-}FL}$	558	155	6 (1)	3.9% (0.6%)
$C_{E\text{-}FL}$ vs. $C_{W\text{-}FL}$	264	78	5 (1)	6.4% (1.3%)
**Combined**	na	203	13 (7)	6.4% (3.4%)
	**Repeated across comparisons**		11 (0)	5.4% (0.0%)
**Within *I. glabra***				
G_{DE} vs. G_{NC}	430	148	--	--
G_{DE} vs. G_{SC}	752	173	5 (2)	2.9% (1.1%)
G_{DE} vs. $G_{E\text{-}FL}$	1175	131	2 (1)	1.5% (0.8%)
G_{NC} vs. G_{SC}	312	185	--	--
G_{NC} vs. $G_{E\text{-}FL}$	722	192	3 (1)	1.6% (0.5%)
G_{SC} vs. $G_{E\text{-}FL}$	424	174	8(4)	4.6% (2.3%)
**Combined**	na	197	10 (4)	5.1% (2.0%)
	**Repeated across comparisons**		4 (0)	2.0% (0.0%)

Table S6. Summary of outlier loci found in host, sex, and geographic comparisons. Posterior probabilities in bold indicate marker found as an outlier in multiple independent population comparisons. Dashes indicate non-significant posterior probabilities (using an alpha of 0.05).

	Between hosts		Within *I. coriacea*		Within *I. glabra*		Between sexes	
**Outlier # (name)**	DFDIST	BAYESCAN	DFDIST	BAYESCAN	DFDIST	BAYESCAN	DFDIST	BAYESCAN
2 (eact.140)	--	--	--	--	--	--	**1**	1
8 (eact.210)	--	--	1	--	--	--	--	--
13 (eact.254.6)	**0.99975**	1	--	--	--	--	--	--
20 (eact.333.8)	--	--	--	--	--	--	**1**	1
22 (eact.349.5)	--	--	--	--	--	--	0.977256	--
28 (eact.392)	--	--	--	--	0.990752	--	--	--
32 (eact.407.4)	--	--	--	--	--	--	**1**	1
41 (eact.457.7)	--	--	--	--	--	--	**1**	1
43 (eact.472.2)	--	--	--	--	--	--	0.979505	--
51 (eact.537)	--	--	0.979505	--	--	--	--	--
70 (eaca.208.1)	**1**	1	0.990502	--	--	--	--	--
72 (eaca.219.3)	**0.99975**	1	0.99975	--	--	--	--	--
74 (eaca.253.3)	--	--	--	--	0.990502	--	--	--
92 (eaca.371.9)	--	--	1	--	--	--	--	--
94 (eaca.388.4)	**1**	1	--	--	--	--	--	--
99 (eaca.404.8)	--	--	--	--	--	--	**1**	--
109 (eaca.469.9)	--	--	--	--	0.986	--	--	--
111 (eaca.489.2)	--	--	0.996751	--	--	--	--	--
113 (eaca.505.8)	--	--	--	--	--	--	0.9915	--
115 (eaca.518.8)	**1**	1	--	--	--	--	--	--
116 (eaca.522.7)	--	--	0.997751	--	--	--	--	--
118 (eaca.532.1)	**1**	1	--	--	--	--	--	--
122 (eaca.584.8)	--	--	0.976256	--	--	--	--	--
124 (eaca.592.8)	--	--	--	--	--	--	**0.99925**	--
125 (eaca.623.9)	--	--	--	--	--	--	**1**	1
132 (eaca.755.4)	--	--	--	--	--	--	**1**	1
137 (eagt.148)	--	--	--	--	--	--	**0.99975**	0.997
144 (eagt.226.3)	--	--	--	0.952	--	--	--	--
148 (eagt.236.3)	--	--	0.984004	--	--	--	--	--
167 (eagt.414.1)	--	--	0.994251	--	0.997001	--	--	--
184 (eagt.552.8)	--	--	--	--	0.995001	--	--	--
188 (eagt.654.5)	--	--	--	--	--	--	0.991252	--
191 (eagt.729.2)	--	--	--	--	--	--	0.990002	--
192 (eagt.737.6)	--	--	--	--	--	--	**1**	1
193 (eagt.739.9)	--	--	--	--	0.993252	--	**1**	0.978
199 (eaga.186.1)	--	--	--	--	--	--	0.993252	--
200 (eaga.210.2)	**0.983754**	--	--	--	--	--	--	--

	Between hosts		Within *I. coriacea*		Within *I. glabra*		Between sexes	
**Outlier # (name)**	DFDIST	BAYESCAN	DFDIST	BAYESCAN	DFDIST	BAYESCAN	DFDIST	BAYESCAN
204 (eaga.249.1)	**0.998**	0.961	--	--	--	--	--	--
213 (eaga.297.7)	**0.994251**	0.989	--	--	--	--	--	--
225 (eaga.402.1)	--	--	0.995001	--	--	--	--	--
226 (eaga.411.3)	--	--	0.99925	--	0.997251	--	--	--
227 (eaga.425.2)	**0.99925**	0.992	--	--	--	--	--	--
229 (eaga.432.4)	--	--	--	--	0.9995	--	--	--
231 (eaga.437.4)	0.993252	0.956	--	--	--	--	--	--
238 (eaga.489.5)	**0.976006**	--	--	--	--	--	--	--
241 (eaga.498.2)	--	--	--	--	0.988503	--	--	--
242 (eaga.499.4)	**0.999251**	0.982	--	--	--	--	--	--
245 (eaga.517.6)	--	--	0.994501	--	--	--	--	--
246 (eaga.518.5)	**0.99925**	1	--	--	--	--	--	--
249 (eaga.542.9)	--	--	--	--	--	--	**1**	1
250 (eaga.543.9)	--	--	--	--	--	--	1	0.999
251 (eaga.583.9)	--	--	--	--	--	--	**1**	1
255 (eaga.651.2)	**1**	1	--	--	--	--	--	--
259 (eaga.672.3)	--	--	--	--	0.993252	--	--	--
260 (eaga.681.6)	--	--	--	--	--	--	**1**	--
261 (eaga.684.6)	--	--	--	--	--	--	**1**	1

Table S7. Distribution of peaks in host-associated outliers. Numbers represent the number of individuals that have a peak at that locus.

**Locus**	**13**	**70**	**72**	**94**	**115**	**118**	**200**	**204**	**213**	**227**	**231**	**238**	**242**	**246**	**255**	**Total**
**Coriacea-flies**																
**VA**	1	0	2	0	2	2	0	0	1	0	1	1	0	2	0	2
**NC**	39	0	41	7	13	44	23	0	38	0	26	26	1	16	0	45
**SC**	34	0	36	4	4	38	11	0	30	0	20	26	0	12	0	38
**East-FL**	4	2	0	0	2	4	0	0	5	0	2	0	1	2	0	5
**West-FL**	4	0	5	0	2	6	3	0	6	0	0	4	0	2	0	6
**Frequency**	0.85	0.02	0.88	0.11	0.24	0.98	0.39	0.00	0.83	0.00	0.41	0.59	0.02	0.35	0.00	
**Glabra-flies**																
**DE**	3	10	0	10	10	0	0	7	1	2	1	0	7	10	5	10
**VA**	0	1	0	0	1	0	0	1	0	0	0	0	1	1	1	1
**NC**	1	20	3	16	24	1	0	11	5	11	1	2	17	24	19	24
**SC**	2	27	2	36	36	1	0	13	10	23	0	4	18	36	34	39
**GA**	1	2	0	3	3	0	0	1	2	2	0	2	2	3	1	3
**East-FL**	1	6	3	8	9	0	0	1	1	4	0	2	2	9	9	9
**West-FL**	0	1	0	1	1	0	0	0	0	0	0	1	0	1	1	1
**Frequency**	0.09	0.77	0.09	0.85	0.97	0.02	0.00	0.39	0.22	0.48	0.02	0.13	0.54	0.97	0.80	

Frequency: the frequency of peaks within the listed host form (coriacea-flies or glabra-flies).
